# Highly Stretchable, Biocompatible, Striated Substrate Made from Fugitive Glue

**DOI:** 10.3390/ma8063508

**Published:** 2015-06-15

**Authors:** Wei Li, Tomas Lucioni, Xinyi Guo, Amanda Smelser, Martin Guthold

**Affiliations:** 1 Department of Physics, Wake Forest University, 7507 Reynolda Station, Winston-Salem, NC 27109, USA; E-Mails: liw210@wfu.edu (W.L.); lucitm12@wfu.edu (T.L.); guox0@wfu.edu (X.G.); 2 Department of Biochemistry and Molecular Biology, School of Medicine Wake Forest University, Winston-Salem, NC 27157, USA; E-Mail: asmelser@wakehealth.edu

**Keywords:** biocompatible substrate, fugitive glue, credit card glue, styrenic block copolymer, stretchable, transparent, fibrin fibers, human mammary epithelial cells

## Abstract

We developed a novel substrate made from fugitive glue (styrenic block copolymer) that can be used to analyze the effects of large strains on biological samples. The substrate has the following attributes: (1) It is easy to make from inexpensive components; (2) It is transparent and can be used in optical microscopy; (3) It is extremely stretchable as it can be stretched up to 700% strain; (4) It can be micro-molded, for example we created micro-ridges that are 6 μm high and 13 μm wide; (5) It is adhesive to biological fibers (we tested fibrin fibers), and can be used to uniformly stretch those fibers; (6) It is non-toxic to cells (we tested human mammary epithelial cells); (7) It can tolerate various salt concentrations up to 5 M NaCl and low (pH 0) and high (pH 14) pH values. Stretching of this extraordinary stretchable substrate is relatively uniform and thus, can be used to test multiple cells or fibers in parallel under the same conditions.

## 1. Introduction

Cells and other biological samples are often exposed to stresses and strains in their natural environment, and these stresses and strains can have a significant biological effect. For example, the growth of smooth muscle cells and the import of nuclear proteins are stimulated by strains [1], and bone formation is stimulated in the presence of mechanical stimuli [2]. In the last few years, strong evidence has emerged that cells are sensitive to the mechanical properties (e.g., stiffness) of their environment. In a seminal paper, Engler *et al.* showed that the differentiation of stem cells is influenced by substrate mechanical properties [3]. These researchers observed that stem cells will differentiate into bone-like cells when grown on stiff substrates or into neuron-like cells when grown on soft substrates. Moreover, mechanical strain plays an important role in stem cell differentiation and function: global gene expression changes (for example, smooth muscle markers increase, cartilage matrix decrease) when cells are aligned parallel to the strain axis [4]. Furthermore, intracellular calcium oscillation in human mesenchymal stem cells is governed by mechanical tension [5] and mesenchymal stem cell differentiation into vascular smooth muscle cells may be promoted by uniaxial strain [6].

Similarly, biological fibers in the body are often exposed to stresses and strains. For example, blood flow exerts stress on fibrin fibers during blood coagulation, and blood flow can affect the structure of blood clots [7,8] and the interaction of fibrin fibers with platelets [9]. Elastin fibers [10], which are found in the skin and lungs, experience strain during respiration and are very extensible; collagen fibers, found in cartilage and connective tissue, experience strain during movement, but are not as extensible [11].

Therefore, when investigating the behavior of biological samples in the lab, a stretchable substrate is required in many situations. There are numerous devices and techniques to apply and test the effect of stress and strains on cells. One example is traction force microscopy, which uses a stretchable substrate. The migration of normal 3T3 (3-day transfer, inoculum 3 × 10^5^ cells) cells can be detected by using traction force microscopy, where the cell body was pulled forward by the dynamic traction forces at the leading edge [12]. Shao *et al.* described a homemade cell stretching device to demonstrate that external mechanical stretch plays a key role in regulating subcellular molecular dynamics with the F-actin cytoskeleton [13]. Moore’s group used their stretching device to show that phenotype modulation (alignment and altered mRNA expression) can be induced by stretching 10T1/2 (from Embryonic mesenchymal cell line) cells [14].

Nano- and microfiber properties are often measured by suspending fibers over microridges in a substrate and then the fibers are manipulated with an Atomic Force Microscope (AFM) [15]. The mechanical properties of different nanofibers have been determined by this method, such as fibrin fibers [15,16], electrospun collagen fibers [17] and electrospun fibrinogen fibers [18]. This sophisticated AFM technique allows for precise mechanical manipulations of nanofibers, and numerous mechanical properties can be extracted with this technique. However, individual fibers are pulled one at a time, which is tedious, time-consuming and not efficient. Varju *et al.* showed that a strained blood clot lyses slower than an unstrained blood clot [19]. So, to investigate the effect of strain on single fibrin fibers, an AFM could be used, but a technique that allows the investigation of multiple fibers in parallel would be more efficient. With our novel, highly stretchable substrate, it is possible to manipulate an array of single fibers simultaneously, instead of only one single fiber. This facilitates investigations of the effect of strain on single biological nanofibers, such as collagen and fibrin, and other nanofibers, such as electrospun nanofibers [18], that are used in biomedical engineering applications.

Besides investigating biological samples, stretchable substrates may also be used in flexible electronics. Stretchable materials can serve as substrates, onto which circuits and electronics are engineered [20,21]. Other times, conducting materials are injected into the substrate [22,23]. Often these materials have a rather low stretch limit, just a little above 100% [24]. However, some applications may require significantly higher elongations. Another drawback of current stretchable devices, like bio-Microelectromechanical systems (bioMEMS) [25,26] and traction force microscopy [12], is the high cost of these devices.

In this paper, we report tests on fugitive glue (a styrenic block copolymer), which is extraordinarily stretchable (up to a 750% strain). It also is very inexpensive, easy to obtain and easy to handle. Moreover, it can be molded into microstructures, and presumably many other shapes. Furthermore, it is transparent (for use in optical microscopy), it can withstand extreme environments, like strong acids/bases (between pH 0 and 14), it is compatible with salt solutions and biological samples (fibrin fibers), and it is non-toxic to cells.

## 2. Results

### 2.1. Stability

#### 2.1.1. Mechanical Stability and Stretchability

We found the substrate to be mechanically stable (it kept its original shape and length) even when stretched to about 250% (detailed experimental conditions are given in Section 4. In our 24-hour test, it kept its original shape in air at room temperature. Therefore, we kept the stretching percentage at around 250% in the following experiments. In the most extreme case we tested, the substrate could be stretched to around 750%, but it was only mechanically stable for 10 min. These experiments demonstrate that substrates formed from fugitive glue are extremely extensible and hold their shapes for long enough time periods to do many biological and other experiments. The extensibility far exceeds that of other common materials used in recently described stretching devices, such as Polydimethylsiloxane (PDMS) [20], Poly-ethylene-terephthalate (PET) [27], Polyimide (PI) [28] and silicone [29]. A concentrated strain of 107% has been reported on a soft, thin PDMS film area in microsupercapacitor arrays [20]. PET substrates coated with an acrylic primer can be stretched to over 70% without breaking [27]. None of these materials can be stretched to several times their original length. In separate experiments, we also tested PDMS (Sylgard 184 Silicone Elastomer Kit, 10:1 mix ratio, Dow Corning, Midland, MI, USA), Norland optical adhesive 81 (Norland Products Incorporated, Cranbury, NJ, USA) and silicone (GE Silicone II, Kitchen and Bath Caulk, clear color, purchased at Home Depot, Atlanta, GA, USA); they were all significantly stiffer than fugitive glue and significantly less extensible.

A familiar example of fugitive glue is its common use to attach credit cards to paper (credit card glue), and it can be easily stretched to several times its original length.

Fugitive glue is a styrenic block copolymer, a type of thermoplastic elastomer. Their mechanical properties, which are similar to rubber, stem from their microstructure. Microscopically, styrenic block copolymers consist of two hard polystyrene end blocks that are connected by a soft, elastomeric midblock (linker), typically made of polybutadiene or polyisoprene.

#### 2.1.2. pH Tolerance Test

**Figure 1:**
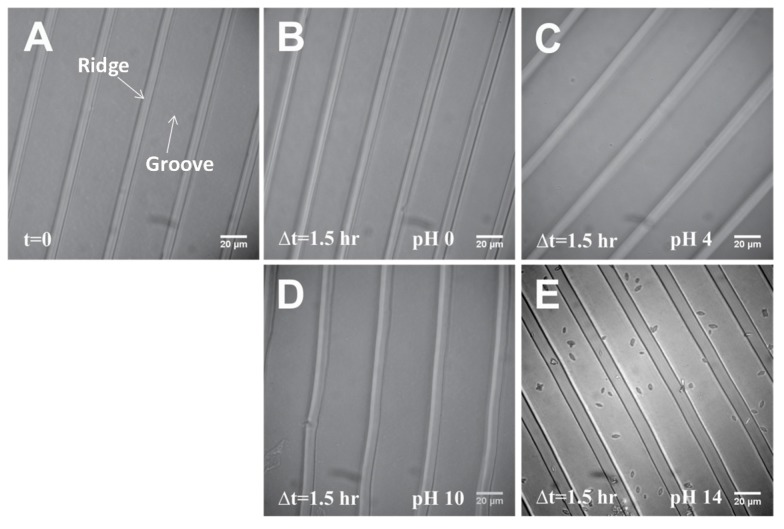
pH Tolerance Test. (**A**) Initial image right after adding pH. (**B**), (**C**), (**D**), (**E**) Images taken after 1.5 h incubation at pH 0, 4, 10, 14. The images before and after adding solutions are at different locations of the same sample. Some crystals formed in the pH 14 solution, probably due to the high Na^+^ concentration, but the substrate appears unaffected by the solution.

Some experiments require extreme pH values, so the ability of a substrate to tolerate highly acidic and basic surroundings can become important. We, therefore, also tested the pH tolerance of our fugitive glue substrate from pH 0 to pH 14. Some polymers may degrade at these extreme environments, e.g., polyanhydrides degrades at high pH values [30], poly(dl-lactide-co-glycolic acid)-methoxypoly (ethyleneglycol) (PLGA-mPEG) microparticles show degradation in strong acid and base [31]. However, our stretchable substrate made from fugitive glue maintained its mechanical and chemical stability under both highly acidic and basic environments for up to at least 1.5 h. There was no discernable degradation (by visual inspection under a microscope with a 40× objective lens) and the shape of the grooves and ridges was not affected (Figure 1).

#### 2.1.3. Salt Solution Tolerance Test

**Figure 2:**
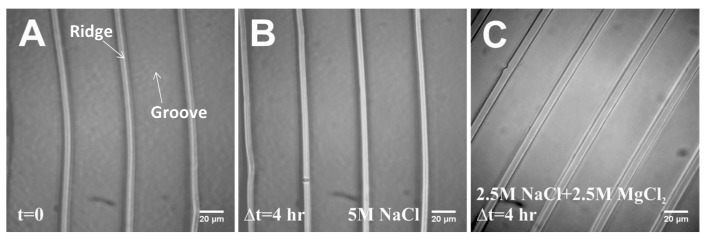
Salt Solution Tolerance Test. (**A**) Initial image that was taken right after adding salt solution to the substrate. (**B**) and (**C**) Images of the substrate after a 4 hour-incubation with 5 M NaCl and (2.5M NaCl + 2.5M MgCl_2_) solutions. The images before and after adding salt solution are at different locations of the same sample.

For biological and non-biological experiments, different salt solutions may be applied to the substrate. So, it is also important to test if this material can withstand different salt solutions. We selected two commonly used salts, NaCl and MgCl_2_, at very high concentrations (near their solubility), reasoning that if fugitive glue can withstand such extremely high salt concentrations, it should also be able to withstand lower salt concentrations. In this experiment, a 5 M NaCl solution and a (2.5 M NaCl + 2.5 M MgCl_2_) solution were applied to the stretchable substrate. After 4 h, no salt deposits and no deformation or degradation of the ridges and grooves were observed (Figure 2).

In summary, the stretchable substrate made from fugitive glue can accommodate a broad range of solution conditions that may be found in many experiments.

### 2.2. Bio-Compatibility

#### 2.2.1. Cell Growth on Fugitive Glue Substrate

**Figure 3:**
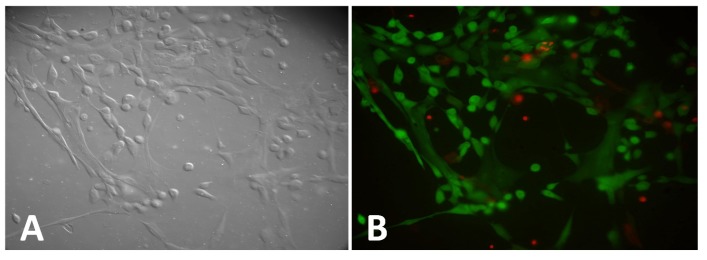
Human mammary epithelial cells grown on fugitive glue substrate. (**A**) Differential Interference Contrast (DIC) images of cells. (**B**) Fluorescence images (20× objective lens) of live cells (stained green) and dead cells (stained red) of same field of view as in (**A**).

Since many biological samples are exposed to stress and strains in their environment, we tested if our stretchable fugitive glue substrate is suitable for biological samples. We tested cells and biological fibers. First, we tested if this material is toxic to cells. Human Mammary Epithelial Cells (HMECs) were grown on fugitive glue for 48 h (Figure 3A,B). Cells were well attached and grew well on the fugitive glue substrate. Thus, it appears that fugitive glue is suitable as a cell substrate. About 85% of cells are alive (stained green), whereas about 15% of cells are dead (stained red).

#### 2.2.2. Fibrin Fiber Formed on Fugitive Glue Substrate

**Figure 4:**
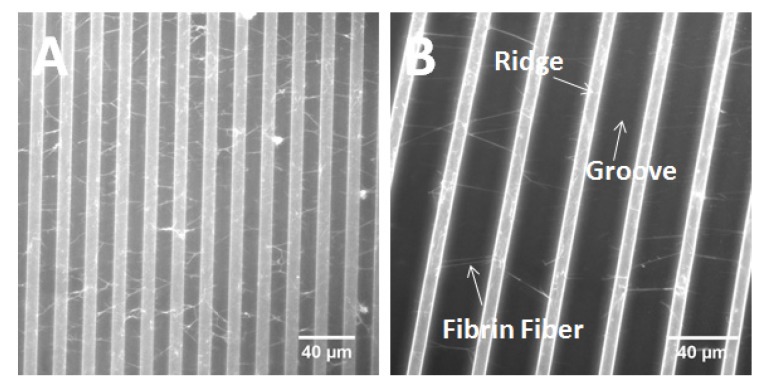
Fibrin fibers on the unstretched substrate (**A**), and stretched substrate (268%) (**B**). The substrate has imprinted ridges and grooves. The width of the groove is 13.5 μm (before stretching), and 36.2 μm (after stretching).

Besides cells, we also tested if our fugitive glue substrate is compatible with biological fibers. Fibrin fibers are the major structural and mechanical component of a blood clot. They have an average diameter of about 130 nm. Fibrin fibers form from fibrin monomers, the activated form of the blood protein, fibrinogen. Fibrinogen gets converted to fibrin by thrombin in the last step of the coagulation cascade. Fibrin fibers can be easily formed in the lab, by adding thrombin to fibrinogen. In previous work, we have determined various mechanical properties of single fibrin fibers, such as their stiffness, extensibility and elasticity [15,16]. As shown in Figure 4A, fibrin fibers form well on this substrate, and they strongly adhere to the substrate. We did not observe any slipping or detachment, even at over two-fold extensions (Figure 4B). Since fibrin fibers experience stress during blood circulation [7,8], there is a strong interest in investigating fibrin fiber mechanical properties. Our stretchable substrate provides a novel approach for these investigations.

## 3. Discussion

We have described and tested a highly stretchable substrate made from fugitive glue, a styrenic block copolymer. It is moldable, transparent to visible light (usable as a substrate in optical microscopy), tolerant to high and low pH values and salt concentrations, and compatible with biological cells and fiber samples. Compared to other devices that are used to apply strain to biological samples on the microscale, it is among the least expensive and easiest to handle and manufacture. Other devices include bio-Microelectromechanical systems (BioMEMS) and some home-made stretching devices.

BioMEMS are MEMS devices for biological applications, which are manufactured using similar microfabrication techniques as those used to create integrated circuits. They are usually used in biosensors, pacemakers, immunoisolation capsules, and drug delivery systems [32]. BioMEMS have been used to apply strain to adherent fibroblasts and detect the de-adhesion force [25], and to test cell force responses: strongly linear, reversible, and repeatable under large stretches [26].

Some home-made devices have also been used to apply external strain to biological systems. For example, Heo *et al.* applied input pressure (air input) from underneath to a PDMS layer, so that the cells on the layer can be stretched [33]. Another novel stretching device is based on the movement of computer-controlled, piezoelectrically actuated pins of a refreshable Braille display underneath a sample to generate strain on a elastometric PDMS membrane’s top surface. The Braille pins could provide 20%–25% maximal strain in the radial direction [34]. Yang’s group used a force sensor probe coated with biomolecules to stretch cells [35]. Wipff *et al.* used PDMS as the elastic membrane, mixed with tracking particles to monitor the degree of substrate expansion under stretch [36].

All these are examples of well-suited devices for biological stretch experiments on a micrometer scale. Many have a limited stretching range (around 20%–30%), since they use PDMS. Our stretchable substrate is a good substrate choice when large strains are required, since it is extremely extensible (about 750%). It is also less stiff than PDMS, and can be stretched manually.

## 4. Experimental Section

### 4.1. Stretchable Substrate Preparation

**Figure 5:**
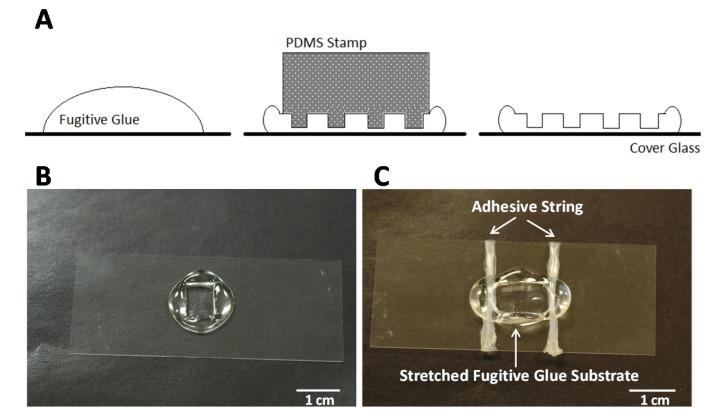
Setup of stretchable substrate. (**A**) Schematic of forming fugitive glue with ridges and grooves; (**B**) Photograph of fugitive glue substrate with ridges and grooves; (**C**) Photograph of stretched substrate.

The stretchable substrate was made from fugitive pressure sensitive adhesive (Surebonder AT-10154 Hot Melt, Hotmelt.com, Edina, MN 55439, www.hotmelt.com); this type of glue is also called hot melt pressure sensitive adhesive or, colloquially, credit card glue. Chemically, this adhesive is a styrenic block copolymer. A drop of hot fugitive glue was placed onto the surface of a microscope cover glass slide (No. 1.5, 24 mm × 60 mm) (Fisherbrand, Pittsburgh, PA, USA) from a Surebonder PRO100 Hot Melt Gun (Hotmelt.com, Edina, MN 55439, www.hotmelt.com). Immediately afterward, a rectangular PDMS (Polydimethylsiloxane) (6 mm × 8 mm) stamp with imprinted grooves and ridges was pressed into the glue. After it cooled down and dried (4 min), the PDMS stamp was peeled off, leaving ridges and grooves in the fugitive glue (width and height of the ridges was 6.5 μm, width of the grooves was 13.5 μm, measured by scanning electron microscope (SEM, Amray 1810, AMRAY, Bedford, MA, USA) in a previous publications [18]. Next, the imprinted fugitive glue substrate was manually stretched to the desired length, as follows. The imprinted substrate was carefully peeled off the glass cover slide, then manually stretched to a specific length, and anchored back down again. For anchoring we used Adhesive Squares ($\frac{1}{2}$ inch × $\frac{1}{2}$ inch Adhesive Squares^™^ RS Industrial, Inc., Buford, GA, USA) as follows. The squares, which are about 1 cm × 1 cm where stretched into a string of about 15 cm. This string was then used to tie down the two sides by wrapping them around the cover glass as shown in Figure 5.

### 4.2. pH and Salt Solution Tolerance Test

400 μL solutions with different pH values and salt concentrations were deposited onto the surface of the stretchable substrate and left for 1.5 h at room temperature. The following solutions were used (all solutions from Fisher Scientific, Pittsburgh, PA, USA): 1 N HCl (pH 0), pH 4 buffer solution (pH-meter calibration solution, Potassium Acid Phthalate), pH 10 buffer solution (pH-meter calibration solution, Boric Acid-Potassium Chloride-Sodium Hydroxide buffer), 1 N NaOH (pH 14), 5 M NaCl (Sigma-Aldrich, St. Louis, MO, USA), 5 M MgCl_2_ (Sigma-Aldrich). Microscope images were taken before and after the tests with an inverted optical microscope (Axio Observer D1, Zeiss, Thornwood, NY, USA) with a 40× objective lens.

### 4.3. Fibrin Fibers

An 18 μL solution of purified human fibrinogen (Enzyme Research Laboratories, South Bend, IN, final concentration 1 mg/mL) was placed onto the surface of the stretchable substrate. Then 2 μL of thrombin (Enzyme Research Laboratories, South Bend, IN, final concentration 0.1 NIH (National Institute of Health) units/mL) were added into it and kept in a wet environment at room temperature for 1 h. After that, a skin (part of the clot) on this solution was peeled off with a pipette tip to reduce the density of the clot before imaging. Fibrin fibers on the stretchable substrate were kept continuously in fibrin buffer (140 mM NaCl, 10 mM, Hepes, 5 mM CaCl_2_, pH 7.4).

### 4.4. Cell Growth

For the cell growth experiments, we used a flat (instead of a striated) substrate made from fugitive glue. Glass bottom dishes (Willcowells, Amsterdam, The Netherlands) of size 35 mm × 2 mm were purchased and assembled in the lab. A drop of hot fugitive glue was placed onto the surface of a petri dish, then a PDMS stamp with flat surface was pressed into the glue and removed after 4 min.

Human mammary epithelial cells (HMECs) were purchased from Lonza (Lonza Group Ltd, Walkersville, MD, USA) and used within 6 passages from their original state from Lonza. HMECs were cultured in Mammary Epithelial Cell Growth Medium–MEGM (Lonza) with 0.4% bovine pituitary extract (BPE) (Lonza), according to the distributor’s recommendations. Cells were cultured and maintained in a culture incubator at 37 ºC with 5% CO_2_. Pictures were taken 48 h after the cells were seeded (Figure 3).

### 4.5. Cell Viability Assay

The cell viability assay was done by using LIVE/DEAD Viability/Cytotoxicity Kit (Life Technologies, Grand Island, NY, USA). 400 μL of 1 μM Calcein AM (acetomethoxy derivate of calcein) (used to stain live cells, ex/em~495 nm/515 nm) and 400 μL of 10 μM Ethidium homodimer-1 (used to stain dead cells, ex/em~495 nm/635 nm) were added into the petri dish with cells prepared as described above and incubated for 15–20 min. Green fluorescence indicated the activity of intracellular esterases present in live cells, and red fluorescence indicated the loss of cell membrane integrity in dead cells. Fluorescence images of live and dead cells and Differential Interference Contrast (DIC) images of cells were taken with a Nikon Eclipse Ti, 20× objective, NA 0.75.
